# Super-Resolution Reconstruction of Remote Sensing Images Using Chaotic Mapping to Optimize Sparse Representation

**DOI:** 10.3390/s24217030

**Published:** 2024-10-31

**Authors:** Hailin Fang, Liangliang Zheng, Wei Xu

**Affiliations:** 1 Changchun Institute of Optics, Fine Mechanics and Physics, Chinese Academy of Sciences, Changchun 130033, China; fanghailin22@mails.ucas.ac.cn (H.F.); xuwei@ciomp.ac.cn (W.X.); 2 University of Chinese Academy of Sciences, Beijing 100049, China; 3 Key Laboratory of Space-Based Dynamic and Rapid Optical Imaging Technology, Chinese Academy of Sciences, Changchun 130033, China

**Keywords:** sparse representation, super-resolution, dictionary learning, chaotic mapping, greedy optimization, remote sensing images

## Abstract

Current super-resolution algorithms exhibit limitations when processing noisy remote sensing images rich in surface information, as they tend to amplify noise during the recovery of high-frequency signals. To mitigate this issue, this paper presents a novel approach that incorporates the concept of compressed sensing and explores the super-resolution problem of remote sensing images for space cameras, particularly for high-speed imaging systems. The proposed algorithm employs K-singular value decomposition (K-SVD) to jointly train high- and low-resolution image blocks, updating them column by column to obtain overcomplete dictionary pairs. This approach compensates for the deficiency of fixed dictionaries in the original algorithm. In the process of dictionary updating, we innovatively integrate the circle chaotic mapping into the solution process of the dictionary sequence, replacing pseudorandom numbers. This integration facilitates balanced traversal and simplifies the search for global optimal solutions. For the optimization problem of sparse coefficients, we utilize the orthogonal matching pursuit method (OMP) instead of the L1 norm convex optimization method used in most reconstruction techniques, thereby complementing the K-SVD dictionary update algorithm. After upscaling and denoising the image using the dictionary pair mapping relationship, we further emphasize image edge details with local gradients as constraints. When compared with various representative super-resolution algorithms, our algorithm effectively filters out noise and stains in low-resolution images. It not only performs well visually but also stands out in objective evaluation indicators such as the peak signal-to-noise ratio and information entropy. The experimental results validate the effectiveness of the proposed method in super-resolution remote sensing images, yielding high-quality remote sensing image data.

## 1. Introduction

Transmission satellites are constrained by the under-sampling effect of imaging sensors and various degradation factors within the imaging link, leading to a prevalent issue of low-resolution remote sensing images. While upgrading hardware is the most direct and effective solution, it carries significant costs and risks. Given the current hardware constraints, obtaining high-resolution images through software using Image Super-resolution Reconstruction (SR) technology presents an effective alternative. Super-resolution, initially derived from the study of natural images, has been extended to remote sensing images. However, remote sensing images encompass a diverse range of ground object types and are subject to numerous degradation factors such as sampling, deformation, blurring, noise in the imaging link, etc. This complexity makes the semantic information of remote sensing images more intricate than that of ordinary images, and obtaining the super-resolution of remote sensing images is even more challenging.

The “Sequence-based Super-Resolution Image Reconstruction Algorithm” proposed by Tsai et al. [1] is a pioneering work in super-resolution research, clarifying that SR technology refers to the technique of converting existing low-resolution (LR) images into high-resolution (HR) images using relevant algorithms. SR technology is dedicated to solving the problem of clearly displaying image textures, structures, edges, and other information after any arbitrary magnification of the image. To a large extent, it meets the demand for high-quality images in fields such as medicine, industry, satellite remote sensing, road monitoring, and security surveillance during the process of societal development. Depending on the methods used in SR, if the interpolation-based methods are not considered, it can be divided into reconstruction-based methods and learning-based methods.

Image reconstruction methods based on reconstruction can be divided into frequency domain methods and spatial domain methods. However, the performance of frequency domain methods is poor and their development is not satisfactory. In contrast, a variety of methods have emerged in the spatial domain. Irani et al. [2] proposed the iterative back-projection approach (IBP) to address the high dependency of super-resolution image reconstruction algorithms on prior image information, but this also makes it impossible to guarantee the uniqueness of the reconstructed images. The projections onto the convex set (POCS) method [3,4] use constraints such as the positivity, boundedness, and smoothness of HR images to preserve the edge information and structural detail information of the reconstructed images, but its computational complexity is high, and its convergence speed is slow. The maximum a posteriori (MAP) estimation method [5] improves image clarity while ensuring the uniqueness of image solutions, but there is room for improvement in extracting image edge information.

Currently, learning-based image reconstruction methods can be divided into pre-deep learning image algorithms and post-deep learning image algorithms. For the pre-deep learning reconstruction algorithms, Freeman et al. [6,7] proposed a single-image reconstruction algorithm based on Markov networks. The neighborhood embedding method [8] extracts image feature information in units of image blocks. Chang et al. [9] significantly reduced the occurrence of overfitting during the model computation process. Sparse representation methods [10,11] focus on dictionary learning and sparse coding as the core to effectively improve image reconstruction efficiency and quality. The Matching Pursuit (MP) algorithm [12] is the earliest method that uses a greedy strategy to provide the approximate solution to sparse representation problems. Based on this, Pati et al. proposed the concept of Orthogonal Matching Pursuit (OMP) [13]. Needell et al. proposed the Regularized Orthogonal Matching Pursuit (ROMP) algorithm [14], Donoho et al. proposed the Segmented Orthogonal Matching Pursuit (StOMP) algorithm in 2012 [15], Sun et al. proposed the Sparse Adaptive Compressed Sampling Matching Pursuit (CSAMP) algorithm [16], Jost et al. proposed a Tree-Based Matching Pursuit (TMP) algorithm [17], and La and Do proposed a new Tree-Based Orthogonal Matching Pursuit (TBOMP) algorithm [18]. In recent years, Tan Jieqing et al. [19] integrated local structural similarity into sparse representation methods, effectively solving the problem of missing image texture structure information caused by traditional sparse representation methods. Shen Yu et al. [20] added a sparse optimization algorithm to the PCA-Net model, which, to some extent, clearly preserved image detail information and edge texture information.

Many scholars have applied deep learning methods to super-resolution problems, enabling Super-Resolution (SR) technology to evolve from the initial small-scale three-layer training model to the current large-scale deep training model. Dong et al. [21] were the first to combine convolutional neural networks with super-resolution image reconstruction techniques, proposing the SRCNN algorithm. Subsequently, FSRCNN [22], ESPCN [23], and multi-scale feature fusion networks [24] were proposed. He et al. [25] stacked multiple residual blocks to form a residual network ResNet. The subsequently proposed models were VDSR [26], EDSR [27], DBPN [28], RCAN, BSRN [29,30,31,32,33,34], etc. Ledig et al. [35] introduced generative adversarial networks into the field of super-resolution reconstruction for the first time, proposing the SRGAN algorithm. Wang et al. [36] further enhanced the visual quality of upsampled images, proposing ESRGAN. GigaGAN [37] introduced filter groups and attention layers into StyleGAN2 [38] and trained the model on billions of images. In recent years, scholars have begun to explore blind image super-resolution networks for unknown degradation patterns [39]. Lee et al. [40] conducted meta-learning from the information contained in the image distribution. DARM proposed a method for learning correction filters adaptively without supervision [41]. Weng et al. first explicitly proposed an overall framework [42]. Blind image SR has certain advantages in reconstructing real-world images with complex degradation types due to the lack of effective SR priors.

Pixel Super-Resolution (PSR) aims to push the resolution beyond the Nyquist sampling limit by similar physical encoding and numerical recovery procedures, bringing it close to the diffraction limit [43]. Recently, PSR phase retrieval has been redefined as a standard optimization problem, allowing the use of off-the-shelf optimization tools such as alternating projection and gradient descent algorithms [44]. However, the current application scope of PSR phase retrieval methods is mainly limited by the substantial time consumption in both the measurement and reconstruction stages. Manual parameter adjustments further complicate data processing.

For remote sensing images, especially those obtained from high-speed imaging systems, the short exposure time results in darker images with significant noise interference. For general super-resolution methods, noise amplification is inevitable during the enlargement process, and further research is needed to determine how to avoid noise influencing this process. The method of sparse representation has inherent robustness in obtaining a noisy super-resolution. Based on this, this paper proposes a sparse representation edge-preserving denoising super-resolution reconstruction method for remote sensing images, named CMOSR, which solves the ill-posed problem of remote sensing images and reconstructs images with a high spatial resolution, good clarity, and rich texture details. This method is of great significance for the further promotion of the theoretical research and practical application of super-resolution reconstruction technology.

The main contributions of the paper are as follows:

1. This paper addresses the issue of poor noise robustness in current super-resolution methods using sparse representation methods that inherently possess noise robustness. It introduces chaotic mapping into the dictionary sequence solving process to optimize the K-SVD dictionary update algorithm.
2. The OMP greedy algorithm, which converges faster than the L1 norm convex optimization algorithm, is adopted to complement K-SVD. Subsequently, a high-resolution image is constructed using the mapping relationship generated by the joint training of high- and low-resolution dictionaries. The local gradient is used as a constraint term to further highlight the high-frequency details of the image and neutralize the smoothness caused during the denoising process.
3. Compared with various super-resolution algorithms that perform well, such as professor Yang Jianchao’s sparse representation method, deep convolution and generative adversarial methods, and pixel super-resolution method, it is concluded that this method can achieve a comprehensive advantage in super-resolution effects for low-resolution remote sensing images in terms of both subjective visual perception and objective evaluation.

The rest of this paper is outlined as follows. Section 2 of this paper is dedicated to elucidating the theory of compressive sensing and its closely related analysis. Section 3 comprehensively expounds on the principles of the methods adopted in this paper. Section 4 describes the methods and experimental results of both subjective and objective analyses. Section 5 discusses the beneficial effects and limitations of the algorithm proposed in this paper. Section 6 concludes the paper, summarizing the main findings and results of the research.

## 2. Analysis of Compressed Sensing Model

### 2.1. Sparse Representation

Remote sensing images contain various pieces of information about the surface, such as the terrain, vegetation, water bodies, etc. In remote sensing images, the distribution of different objects or scenes is usually uneven; some areas may have higher grayscale or color values, while others are lower or close to zero. This uneven distribution gives remote sensing images a certain degree of sparsity. Compressed sensing theory proposes a new data acquisition method based on signal sparsity, using sparse optimization algorithms to achieve the high-precision reconstruction of signals, breaking through the classic sampling theorem.

The overcomplete dictionary learning discussed in this paper is essentially sparse coding. The principle involves treating images as a special type of signal, selecting the least possible linear combinations of basic signals from an ever-updating overcomplete dictionary to express the original signal as much as possible. The coefficients for linear representation are referred to as sparse coefficients. Signals can be reconstructed from the dictionary and sparse representation coefficients. Essentially, images have similar structural representation elements, and these structures are high-frequency information, such as image boundary information, texture structure information, etc. Certain structural components in the image constitute useful information that can be represented by atoms. However, noise in the image is randomly distributed and lacks structural features, so it cannot be represented by atoms. Therefore, the sparse reconstruction process can separate the useful information from the noise in the image, thereby achieving the purpose of image denoising.

**Figure 1:**
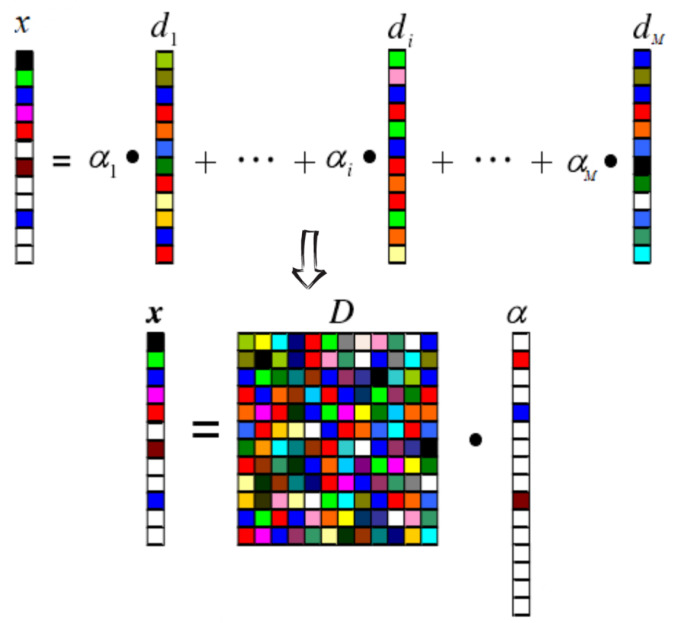
Schematic diagram of sparse representation principle.

The principle of sparse representation is illustrated in the Figure 1. The formula is expressed as follows:

(1) $$x_{x\in R^{N}}=D\cdot \alpha =\underset{D\in R^{N\times M}}{\underset{︸}{\left(d_{1}\left|\cdots \right|d_{i}\left|\cdots \right|d_{M}\right)}}\underset{\alpha \in R^{M},sparse\;coefficients}{\underset{︸}{{\left(\alpha_{1},\alpha_{2},\cdots ,\alpha_{M}\right)}^{T}}}$$

where x denotes the N × 1 dimensional original signal, $D=\;\left[d_{1},...,d_{i},...,d_{M}\right]\in R^{N*M}\left(N<M\right)$ denotes the N × M dimensional overcomplete dictionary, and $\alpha =\;{\left[\alpha_{1},...,\alpha_{i},...,\alpha_{M}\right]}^{T}\in R^{M}$ denotes the M × 1 dimensional sparse matrix.

### 2.2. Information Reconstruction

In the process of information reconstruction and recovery at high frequencies, noise is also amplified simultaneously. Designing an appropriate reconstruction algorithm is a crucial step in ensuring the quality of the output image. The noise components in the whole link of remote sensing imaging are complex, there are many noise interference sources, and the noise components with different distribution and statistical characteristics are difficult to effectively suppress by a single denoising algorithm. In an optical remote sensing image, the effective signal can be regarded as a sparse signal, while the noisy signal can be regarded as a random and non-sparse signal. Through the sparse representation, the sparse components in the image can be extracted to reconstruct the image, and the noise will be regarded as the residuals between the original image and the reconstructed image, and the purpose of noise reduction can be achieved by removing the residuals. Information that has been sparsed needs to be decoded before the final target information can be obtained. Since m < n, the linear equation corresponding to compressed sensing is an underdetermined equation, which can have either no solution or infinitely many solutions. This makes the problem of compressed sensing ill-posed. Excluding the case of no solution, the core issue of the reconstruction algorithm for compressed sensing is to find the specific solution needed from among infinitely many sets of solutions. In addition to satisfying the conditions of the linear equation, it is necessary to add specific constraints to select this particular solution. Based on different mathematical models and data optimization methods, a variety of algorithms have emerged. Commonly used compressed sensing algorithms include iterative reconstruction algorithms based on L1 norm minimization, reconstruction algorithms based on sparse representation, compressed sensing algorithms based on image blocks, etc. The Orthogonal Matching Pursuit (OMP) converges faster than the convex optimization method based on the L1 norm in solving such hard non-convex problems, enabling rapid high-quality signal reconstruction, making it suitable for high-speed imaging systems.

(2) $$\left\{\tilde{D},\tilde{\alpha }\right\}=\arg\min\left\{{∥x-D\alpha ∥}_{F}^{2}+\sigma {∥\alpha ∥}_{1}\right\}$$

The application of sparse dictionary learning to image reconstruction includes two important steps: dictionary training and sparse reconstruction. Assuming that the input low-resolution image blocks can be sparsely linearly expressed by the training sample set, the high- and low-resolution dictionaries are obtained through training and learning a large number of images similar to the target in the process of dictionary training. By adopting the joint dictionary training method, the high- and low-resolution image blocks under the dictionary will have the same sparse representation coefficients. Therefore, during reconstruction, the sparse coefficients under the low-resolution dictionary combined with the high-resolution dictionary can yield a high-resolution reconstructed image.

## 3. Proposed Method

**Figure 2:**
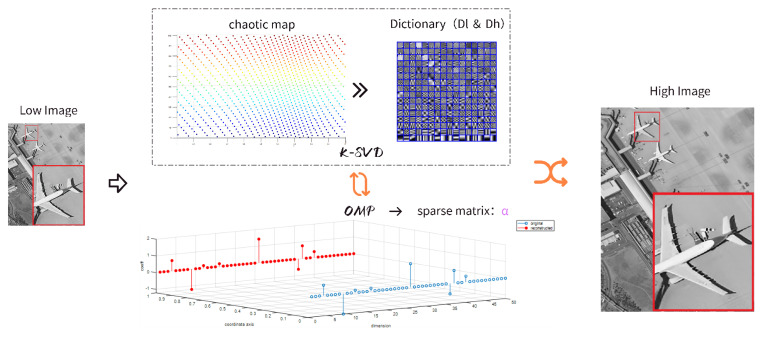
Illustration of proposed model.

This paper proposes a method for 2× super-resolution reconstruction using edge-preserving denoising with sparse representation optimized by chaotic mapping. Chaotic mapping can be used as an alternative to pseudorandom number generators to generate chaotic numbers between 0 and 1, often with better results than pseudorandom numbers. It has good distribution and randomness, and the distribution density of the results presented by the map is relatively uniform. Circle chaotic mapping is relatively stable, has high coverage of chaotic values, and is more widely used in the field of image processing, so this paper chooses circle chaotic mapping to optimize the sparse dictionary. The illustration of the proposed model is shown as Figure 2. The low-resolution noisy remote sensing image, Low Image, is used as the target of this algorithm. The K-SVD dictionary learning algorithm is optimized through circle chaotic mapping, resulting in an overcomplete joint dictionary containing pairs of high- and low-resolution dictionaries. The purpose of the joint training of high-resolution and low-resolution image blocks is to have the same representation coefficient α on their corresponding over-complete dictionaries, so as to reduce the complexity of the process of reconstructing high-resolution images. For the low-resolution image features to be reconstructed, only the sparsity coefficient under the low-resolution dictionary Dl is required, and then, combined with the high-resolution dictionary Dh, the high-resolution image features can be reconstructed. In this process, the OMP algorithm and K-SVD complement each other, updating to obtain the corresponding sparse coefficient.

### 3.1. Dictionary Update and Sparse Coefficient Solution

In the actual super-resolution process, the initial sparse coefficients are obtained by OMP and the initialized dictionary. Here, the initial dictionary D is based on a learning model, not on an analytical model (pre-constructed), such as using a Fourier base, a DCT base, a Gabor base, etc. The dictionary obtained by dividing the input image into small patches is highly adaptable and can capture the geometric features of the image. Subsequently, the joint training update of high- and low-resolution dictionary pairs is completed using the K-SVD algorithm. That is, from the high- and low-resolution training dataset, the high- and low-resolution dictionary pairs are trained, and the objective function is solved as follows; the selection of hyperparameters in the formula is referred to the relevant literature [45,46]:

(3) $$\left\{\tilde{D},\tilde{\alpha }\right\}=\arg\min\left\{{∥x-D\alpha ∥}_{F}^{2}+\sigma {∥\alpha ∥}_{1}\right\}s.t.{∥\alpha ∥}_{0}\leq T_{0}$$

where $\tilde{D}$ is the updated overcomplete dictionary; $\tilde{\alpha }$ is the updated sparse matrix; $\|{\|}_{F}$ denotes the F-norm, $\|{\|}_{1}$ denotes the 1-norm, $\|{\|}_{0}$ denotes the 0-norm; σ denotes the regularization term influence factor; $T_{0}$ denotes the upper limit of the non-zero sparse representation coefficient vector.

α is obtained from the OMP and the model can be simplified as

(4) $$\tilde{D}=\arg\min\left\{{∥x-D\alpha ∥}_{F}^{2}+\sigma {∥\alpha ∥}_{1}\right\}$$

Set the number of iterations, *k*, to be the *k*th column vector of the dictionary D to be trained, and update the dictionary column by column; all columns need to be updated, and when updating to a certain column, the other columns of the dictionary are fixed.

(5) $$\begin{array}{cc} {∥\begin{array}{c} x-D\alpha \end{array}∥}_{F}^{2} & ={∥x-\sum_{j=1}^{k}d_{j}\alpha_{T}^{j}∥}_{F}^{2}\\ \; & ={∥\left(x-\sum_{j\neq k}d_{j}\alpha_{T}^{j}\right)-d_{k}\alpha_{T}^{k}∥}_{F}^{2}\\ \; & ={∥E_{k}-d_{k}\alpha_{T}^{k}∥}_{F}^{2} \end{array}$$

where $d_{j}$, $\alpha_{T}^{j}$ denotes the status updated to the *j*th column.

(6) $$E_{k}=x-\sum_{j\neq k}d_{j}\alpha_{T}^{j}$$

Since the residuals are not directly solvable, the optimization problem is deformed into

(7) $$\min∥E_{k}\Omega_{k}-d_{k}\alpha_{T}^{k}\Omega_{k}{∥}_{F}^{2}=\min{∥E_{k}^{R}-d_{k}\alpha_{R}^{k}∥}_{F}^{2}$$

where $\Omega_{k}$ is an N × $\left|W_{k}\right|$ matrix whose elements are 1 at positions ($W_{k}$(i), i) and 0 at all other positions, and the set $W_{k}$ is the set consisting of the indices of all signals used in the signal decomposition.

The SVD decomposition can be performed:

(8) $$E_{k}=U\Delta V^{T}$$

where the first column of U is the optimization of $d_{k}$.

Update sequentially until a new dictionary $\tilde{D}$ (${\tilde{D}}_{L}$ and ${\tilde{D}}_{H}$ ) is generated. Get the latest sparse matrix $\alpha_{T}^{k}$.

The process of the K-SVD algorithm dictionary updating involves the problem of dictionary modeling, and the algorithm in this paper innovatively introduces circle chaotic mapping to optimize the process of dictionary sequence solving. Its formula is as follows:

(9) $$x_{i+1}=\mathrm{mod}\left(x_{i}+b-\left(\frac{a}{2\pi }\right)\sin\left(2\pi x_{k}\right),1\right)$$

where a and b are control parameters with the commonly used values of 0.5 and 0.2.

OMP solves the sparse matrix α as follows:

Initialize sparse matrices.

(10) $$\alpha =\vec{0}$$

Initialize the residual vector.

(11) $$r_{0}=x$$

Initialize the index set to the empty set.

(12) $$\Lambda_{0}=\emptyset$$

Select the index *i* in the dictionary D corresponding to the atom with the largest projection of the residual *r*.

(13) $$\begin{array}{cc} \arg\max_{i} & =\left(\left|\langle d_{i},r\rangle \right|\right)\\ & \forall i\notin \Lambda \end{array}$$

where $\langle d_{i},r\rangle$ denotes the inner product of the dictionary atoms and residuals. Find a subdictionary in dictionary D that corresponds to the index set.

(14) $$D_{t}=D_{t-1}\cup d_{i}$$

Use linear regression to solve for the optimal sparse vector $\hat{\alpha }$ such that the least squares approximation to the residual *r*.

(15) $$\hat{\alpha }\;[\Lambda_{t}]=D_{t}^{+}x,D_{t}^{+}={\left(D_{t}^{H}D_{t}\right)}^{-1}D_{t}^{H}$$

Update the residuals.

(16) $$\begin{array}{cc} r_{t} & =x-D_{t}\hat{\alpha }\\ & =x-D_{t}\left(D_{t}^{+}x\right) \end{array}$$

(17) $$D_{t}^{+}={\left(D_{t}^{T}D_{t}\right)}^{-1}D_{t}^{T}$$

where $D_{t}^{+}$ is the pseudo-inverse of $D_{t}$.

Judge the termination condition and end if *t* > sparsity K Output $\hat{\alpha }$.

Synthesize the denoised signals.

(18) $$x_{denoise}={\tilde{D}}_{L}\hat{\alpha }$$

${\tilde{D}}_{L}$ is obtained by the K-SVD dictionary updating algorithm.

After that, it will be interpolated and enlarged to the same size of the target high-resolution image and cut into a number of overlapping small blocks of the same size as the atomic size of $D_{L}$ using the previous algorithm to solve for the sparsity coefficient α, combined with $D_{H}$ and converted into a number of high-resolution small blocks, which are averaged over the overlapping blocks to obtain HIm.

(19) HIm=D˜Hα^

### 3.2. Local Gradient Optimization

**Figure 3:**
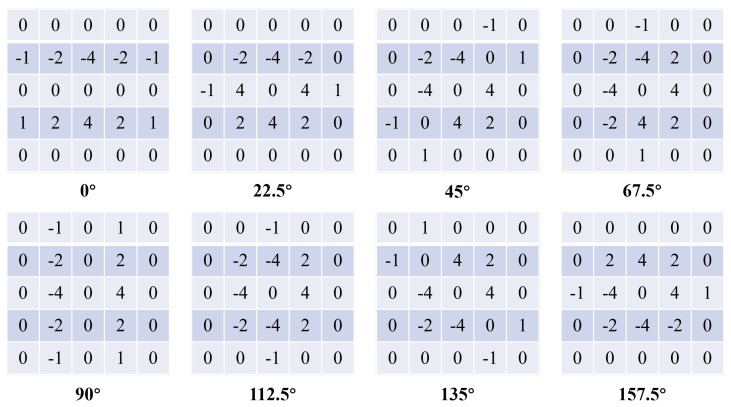
Eight-direction Sobel operator.

The Sobel edge detection operator is used to make the detected edges be relatively fine to optimize the local gradient of the obtained image HIm, so as to obtain a clearer edge image H˜Im. The algorithm uses an improved eight-direction Sobel operator instead of the classical Sobel operator with only two directions, horizontal and vertical, which can better detect edges in different directions, as shown in Figure 3.

(20) H˜Im=HIm+λ·G

The pseudocode of the algorithm in this paper is shown in Algorithm 1 below.
**Algorithm 1** Super-Resolution Reconstruction of Remote Sensing Images Based on Sparse Representation.1. Input: noisy low resolution image *x*, initial dictionary $D_{L}$parameter settings: σ,λ, kmaximum number of iterations k, residual *r*2. Initialization: initial dictionary $D_{L}=D_{0},D_{H}=D_{0}$3. while n<k4. for image x, do$\left\{\tilde{D},\tilde{\alpha }\right\}=\arg\min\left\{{∥x-D\alpha ∥}_{F}^{2}+\sigma {∥\alpha ∥}_{1}\right\}$$s.t.{∥\alpha ∥}_{0}\leq T_{0}$5. SVD decomposition $E_{k}=U\Delta V^{T}$6. update the dictionary $\tilde{D}$7. chaotic mapping $x_{i+1}=\mathrm{mod}\left(x_{i}+b-\left(\frac{a}{2\pi }\right)\sin\left(2\pi x_{k}\right),1\right)$8. update $r_{t}=x-D_{t}\hat{\alpha }=x-D_{t}\left(D_{t}^{+}x\right)$9. end10. end11. Output ${\tilde{D}}_{L},{\tilde{D}}_{H},\tilde{\alpha }$

## 4. Experiments

In this section, the performance of the proposed method is evaluated. First, we present the experimental empirical parameters of the system. Second, we compare the performance of the proposed algorithm (CMOSR) with other representative algorithms for the 2× super-resolution images and analyse the results obtained from the comparison. In order to fully evaluate the method proposed in this paper, all experiments were performed on MATLAB R2024a and the programs were run on a Windows 11 server with 16 GB of RAM and a 3.2 GHz CPU. The image data include laboratory target maps, the NWPU VHR-10 dataset [47], and the FAIR1M1.0 dataset [48]. In this experiment, after carefully studying the literature for similar methods [10,11,13] as well as experimental testing and validation, the empirical values for the relevant parameters are defined as follows: σ at 8, λ set to 0.2. It is worth noting that the algorithm in this paper is an improvement of the related classical sparse compressive sensing algorithms, so the experiment is not complicated, and the original codes involved in the literature given in the paper can be referred to; the original method can be replaced according to the specific scheme proposed by this paper’s algorithm. When the value of σ is over 10, the image distortion after over-scoring is serious, and the details of the contours of the feature information are lost. On the contrary, when the value is under 5, the decontamination and denoising effect of the image is not obvious. When the value of λ is over 1, the image is over-smoothed, and the details become blurred. On the other hand, when the assumed value is under 0.05, the effect of the local gradient as a constraint term is too weak. In this paper, CMOSR is compared with the sparse representation image super-resolution algorithm proposed by Jianchao Yang(ScSR), SRCNN, EDSR, SRGAN, the pixel super-resolution algorithm (PSR), and the GigaGAN AI-based super-resolution method. Because the deep learning algorithms require a long pre-training time, and the proposed algorithm does not require a pre-training process, it is impossible to compare the time at a fair level. However, it is worth mentioning that the algorithm in this paper takes less than one minute to process 256 × 256 remote sensing images on a graphics workstation with NVIDIA GTX1660 5991MB, 32GB of RAM and about two minutes to process 512 × 512 remote sensing images. Combined with engineering experience, this time is sufficient for the application.

### 4.1. Comparison of Denoising Effects

**Figure 4:**
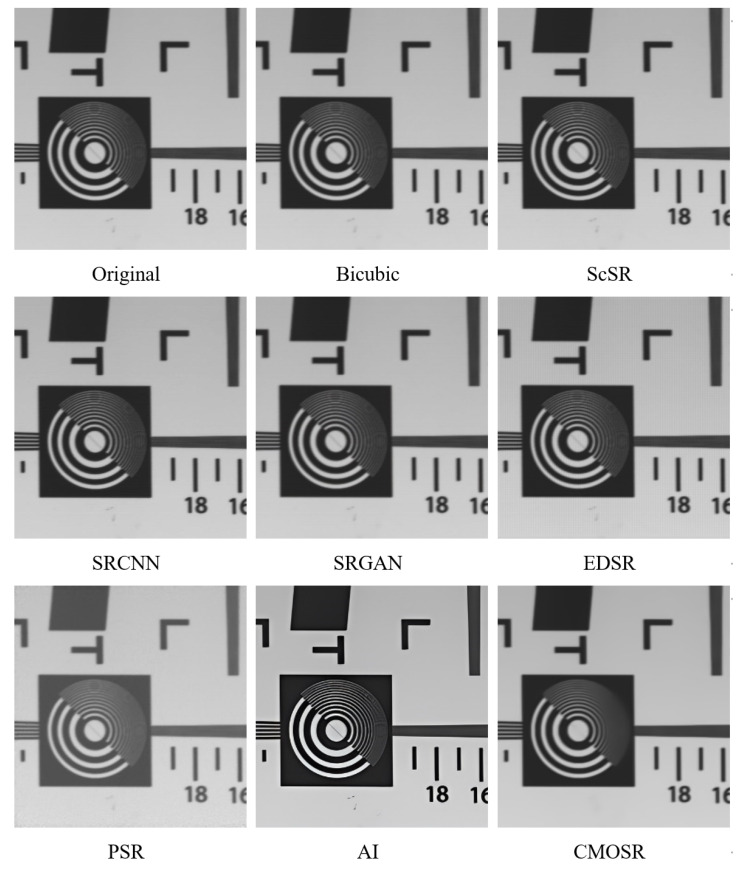
Comparisons of decontamination effect of target image.

Target images are an important class of images in the radiation calibration experiment of space cameras. In order to fit the engineering significance of this algorithm more closely, the target image actually taken by the space camera in our laboratory is selected. As shown in Figure 4, the original image contains multiple stains. The images obtained after reaching super-resolution using methods such as ScSR and deep learning methods like SRCNN still contain stains. The image obtained by PSR has low contrast. The image obtained by the GigaGAN AI method has sharp and clear edges, but the stains still exist. However, when we processed it with CMOSR, all stains disappeared completely, and the image was clear and visually appealing, although accompanied by a degree of smoothing.

**Figure 5:**
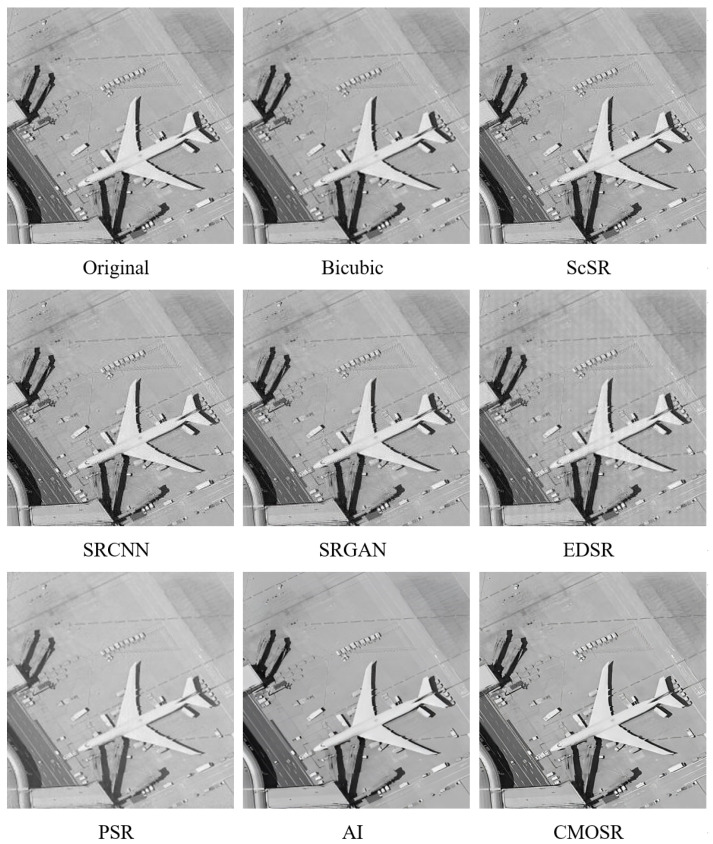
Comparisons of aircraft fuselage denoising effect.

As shown in Figure 5, the original image is severely contaminated by noise points, especially and noticeably on the aircraft body. The noise in the image using ScSR not only remains but tends to increase. Neither the SRCNN nor SRGAN algorithms have a good suppression effect on noise. Artifacts are visible in the EDSR-processed image. PSR has a certain effect on improving the noise in the original image, and GigaGAN AI and CMOSR perform well, basically filtering out all the noise.

### 4.2. Comparison in the Sharpness of the Edges

**Figure 6:**
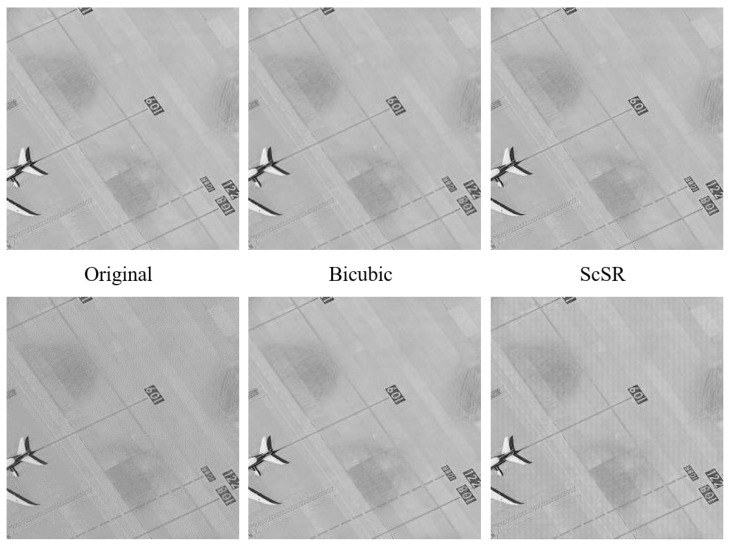
Comparisons of edge clarity effect of airport ground marking.

In the following Figure 6, mainly for the comparison of digital signs on the ground, it can be seen that there is obvious noise around it, and there is bubble-like pollution in SRCNN and obvious artifacts in EDSR. ScSR and SRGAN do not inhibit the contamination of the edge of the logo, the PSR performs decently, and the super-resolution image obtained by the GigaGAN AI algorithm is clear and high-contrast, but has the disadvantage of losing authenticity. The image obtained by CMOSR in this paper has more clear identification edges.

**Figure 7:**
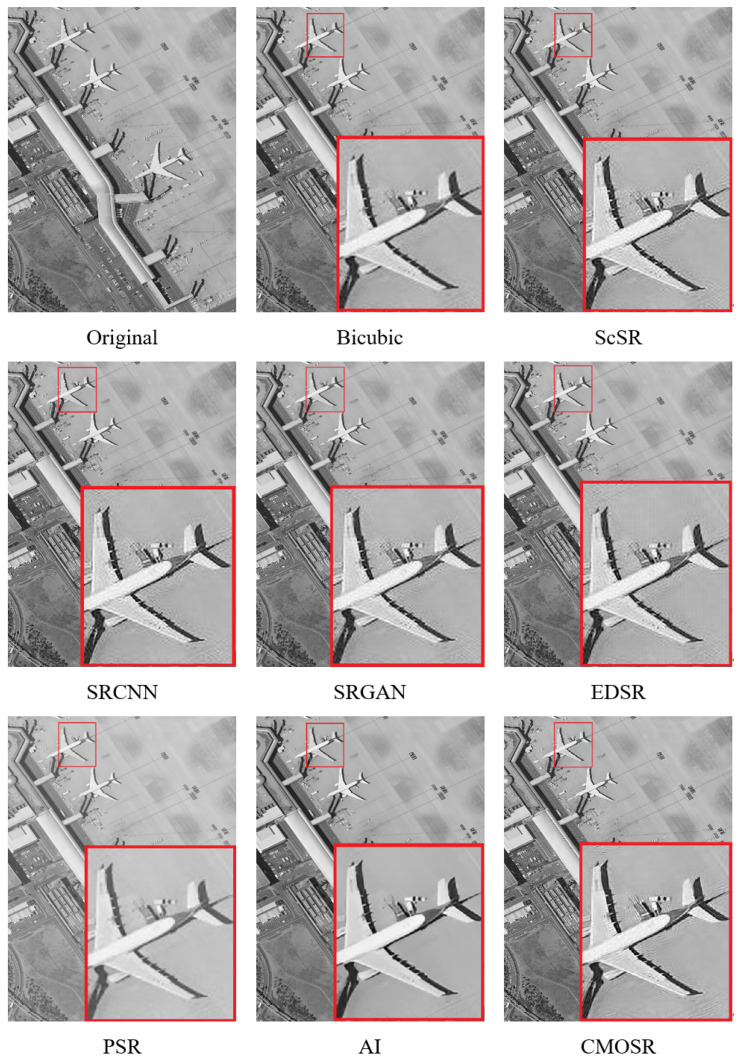
Comparisons of signal-to-noise ratio and local distortion level of remote sensing images.

As shown in Figure 7, the comparisons are mainly focused on the degree of local distortion in the images obtained after attaining a super-resolution by various algorithms. The images obtained by ScSR and a number of deep learning methods exhibit multiple distortions and have poor visual quality. From both global and local perspectives, PSR, GigaGAN AI, and CMOSR can all yield images of good quality.

### 4.3. Comparison of Objective Indicators

The Peak Signal-to-Noise Ratio (PSNR) and Structural Similarity Index (SSIM) are the most commonly used metrics for evaluating image reconstruction quality. The formulas are as follows:

(21) $$\mathrm{MSE}=\frac{1}{m}\sum_{i=1}^{m}{\left(y_{i}-\hat{y_{i}}\right)}^{2}$$

(22) $$\mathrm{PSNR}=10\times lg\left(\frac{{\mathrm{MaxValue}}^{2}}{\mathrm{MSE}}\right)$$

where MSE is the mean square error of two images; MaxValue is the maximum value that can be taken by an image pixel.

The SSIM (structural similarity) is also a full-reference image quality evaluation metric, which measures image similarity in terms of brightness, contrast, and structure, respectively.

(23) $$\mathrm{SSIM}\left(x,y\right)=\frac{\left(2\mu_{x}\mu_{y}+c_{1}\right)\left(2\sigma_{\mathrm{xy}}+c_{2}\right)}{\left(\mu_{x}^{2}+\mu_{y}^{2}+c_{1}\right)\left(\sigma_{x}^{2}+\sigma_{y}^{2}+c_{2}\right)}$$

where $\mu_{x}$ is the mean of x; $\sigma_{x}$ is the variance of x; $\sigma_{\mathrm{xy}}$ is the covariance of x and y; $c_{1}={\left(k_{1}L\right)}^{2},c_{2}={\left(k_{2}L\right)}^{2}$ are constants to prevent division by zero (L is the range of pixel values); $c_{3}=c_{2}/2$; x and y are N × N images of the same size.

The FSIM (Feature Similarity Index Measure) is a metric used to assess the quality of an image. The FSIM measures the similarity between images mainly based on the similarity between perceived features. It captures the structural information of an image from a perceptual point of view and is highly correlated with the evaluation of image quality as subjectively perceived by humans and can be used in applications such as image noise reduction, super resolution reconstruction, and image enhancement.

(24) $$\mathrm{FSIM}=\frac{\sum_{x\in \Omega }S_{L}\left(x\right)\cdot PC_{m}\left(x\right)}{\sum_{x\in \Omega }PC_{m}\left(x\right)}$$

Phase congruency (PC) can be a good way to portray local structure. At the same time, because PC has relative invariance to image changes, which is conducive to the extraction of stable features in the image, but, sometimes, the changes in the image do affect the perception, we need to use the gradient magnitude (GM) to make up for the gradient features. The FSIM uses two features, PC and GM, to complement each other.

The similarity of PCs is calculated as follows:

(25) $$S_{PC}\left(x\right)=\frac{2PC_{1}\left(x\right)\cdot PC_{2}\left(x\right)+T_{1}}{PC_{1}^{2}\left(x\right)+PC_{2}^{2}\left(x\right)+T_{1}}$$

The GM similarity is calculated as follows:

(26) $$S_{G}\left(x\right)=\frac{2G_{1}\left(x\right)\cdot G_{2}\left(x\right)+T_{2}}{G_{1}^{2}\left(x\right)+G_{2}^{2}\left(x\right)+T_{2}}$$

The similarity of PC and GM fusion is as follows:

(27) $$S_{L}\left(x\right)={\;[S_{PC}\left(x\right)]}^{\alpha }\cdot {\;[S_{G}\left(x\right)]}^{\beta }$$

In information theory, entropy is used to describe the richness of information. The information entropy evaluation function is based on the distribution of image grayscale values. When the pixel grayscale value distribution range is wide and there is a large difference between grayscale values, the entropy value is high. Based on information entropy, a clarity evaluation function is constructed, defined as follows.

(28) $$F=-\sum_{g=0}^{G}P_{k}\left(g\right)\log_{b}P_{K}\left(g\right)$$

where b is generally taken as 2, g denotes the grey value of the image, G denotes the maximum of the grey value of the image, k denotes the sequence of images, and $P_{K}\left(g\right)$ denotes the probability of the occurrence of the grey value g in the kth image:

(29) $$P_{k}\left(g\right)=\frac{n_{g}}{MN}$$

where MN represents the total number of pixels and $n_{g}$ denotes the number of pixels with the grey value g in the kth image.

A higher signal-to-noise ratio (SNR) indicates clearer and more distinguishable signals.

(30) $$SNR=20\mathrm{lg}\frac{mean}{rmse}$$

where lg denotes the logarithm of the base 10. Calculate the variance of the entire image or a specific region as a measure of noise. Divide the root mean square (standard deviation) of the noise by the mean value of the signal to get the signal-to-noise ratio.

**Figure 8:**
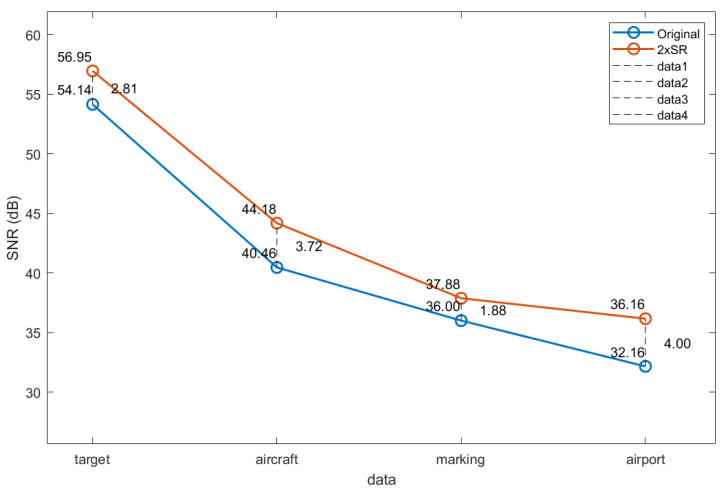
Line graph of SNR changes.

Since the super-resolution object of this algorithm is mainly for the faint and low-quality images obtained by the high-speed space camera in our laboratory, the comparison of the images before and after obtaining a super-resolution is very necessary. To further measure the noise filtering effect of the proposed algorithm, a uniform area of 50 × 50 pixels was selected from each of the four images for signal-to-noise ratio tests before and after reaching a super-resolution. The results are plotted as a line graph in Figure 8, and it can be concluded that after obtaining a super-resolution by CMOSR, the signal-to-noise ratio can be increased by an average of 2–4 dB.

**Figure 9:**
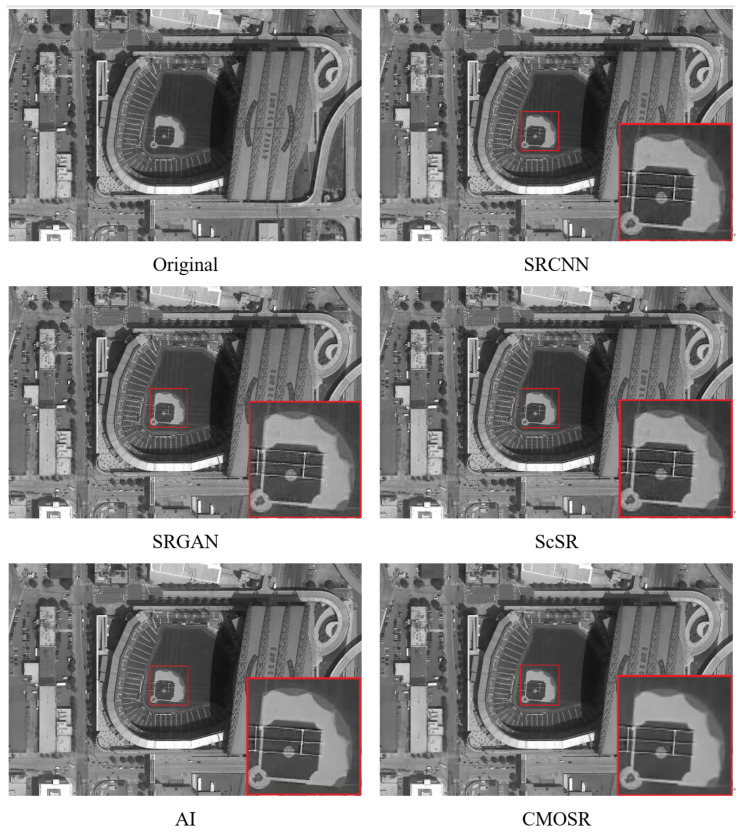
Comparisons of image super-scoring effect of stadium.

**Figure 10:**
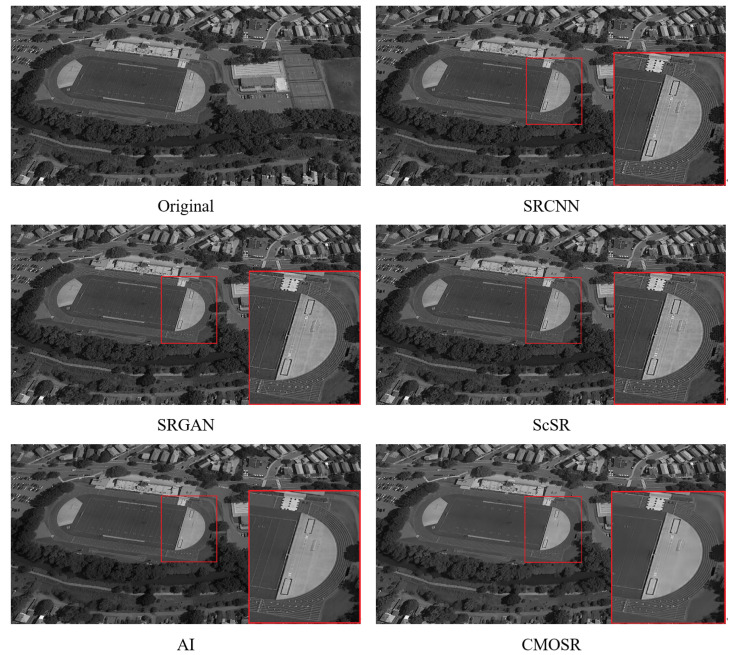
Comparisons of super-scoring effect of football field images.

After the comparative experiments mentioned above, it can be seen that CMOSR, as proposed in this paper, has a certain competitiveness. Since the algorithm is mainly used for large-scale remote sensing images, additional experiments will continue with the SRCNN, SRGAN, ScSR, and GigaGAN AI algorithms based on GigaGAN, which performed well in the previous experiments. The experiment selected images of a sports stadium and football field taken by space cameras from a ground view and displayed the super-resolution images of each algorithm after local magnification; the results are shown in Figure 9 and Figure 10.

**Figure 11:**
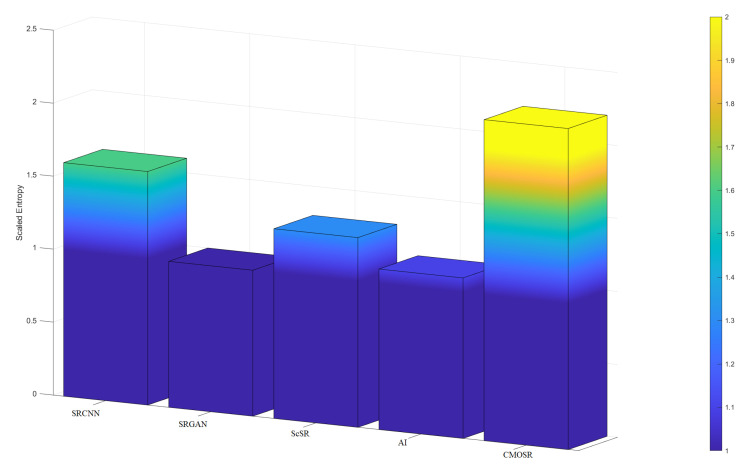
Comparisons of scaled entropy values of stadium.

**Figure 12:**
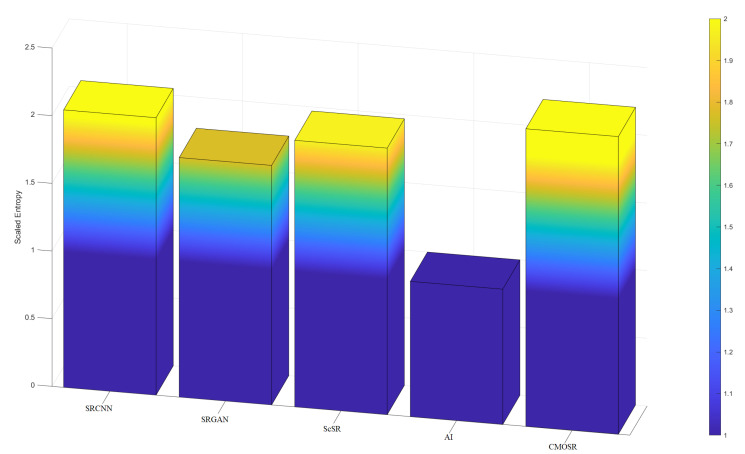
Comparisons of scaled entropy values of football stadium.

As can be seen from the comparison tables of indicators (Table 1, Table 2, Table 3 and Table 4), the PSNR value of the images obtained by CMOSR are generally leading compared with others obtained by other methods. Since noise also belongs to the structural components of the original image, the SSIM and FSIM of the image filtered by CMOSR remain over 0.9 and near 0.99, leading the pack of comparative algorithms. By comparing the information entropy evaluation function values of super-resolution images obtained by various methods, it is known that CMOSR can obtain images with higher information entropy in most cases, compared with other methods. To more intuitively demonstrate the superiority of CMOSR in terms of image information richness compared with other algorithms, bar charts based on entropy values (Table 5 and Table 6) are drawn as shown in Figure 11 and Figure 12. To enhance the presentation, the scaled entropy value is derived by multiplying the difference between each set of entropy values and the minimum value in that set by a certain factor. It can be seen that the stadium and football stadium images super-resolved by the method proposed in this paper both possess the highest quality of information richness. The pseudorandom numbers method is used in ScSR, so it can be seen that circle chaotic mapping can achieve better results in image reconstruction than pseudorandom numbers. In addition, during the above experiments, we found that the images processed by the deep learning algorithm have false details and even some stripe-like artifacts, which are not included in the original image, so we do not want to retain these contents. The image processed by the pixel method becomes lighter in color to a certain extent, and the size of the image is more stringent, and the rows and columns must be even-numbered pixels. The proposed algorithm does not need to be trained on a large number of remote sensing images in advance and does not generate some unexpected details when processing images and is more inclusive of the image size, which is more universal in the field of remote sensing image processing with strict content requirements. In summary, the images reconstructed after obtaining a super-resolution by CMOSR have higher quality and good authenticity.

## 5. Discussion

### 5.1. Results Discussion

CMOSR is mainly proposed under the engineering background that the remote sensing images obtained by space cameras are usually weak. Therefore, the effect to be achieved is actually different from the ordinary super-resolution reconstruction algorithms. Especially for the high-speed space cameras developed in our laboratory, obtaining the super-resolution of remote sensing images should be prioritized: we pay more attention to the main information in the image so that it can be better identified, rather than reconstructing all the information in high quality. As shown in the Section 4, we would prefer that key pieces of information such as the aircraft and site identification be seen more clearly, rather than background information such as unimportant lines on the ground. Through comparative analysis experiments between CMOSR and other existing methods in terms of subjective and objective aspects, it is clear that the CMOSR proposed by us can obtain high-quality images with good perception and no loss of authenticity, whether in terms of removing image stains or edge sharpening. The PSNR value is basically at the highest level, and although the SSIM is not at the highest, it also reaches a relatively high value. The removal of noise as structural information in the original image inevitably leads to a decrease in the index, which, to some extent, can demonstrate its ability to filter out noise. In terms of the information entropy, a measure of image information richness, CMOSR performs better than other algorithms. This result can be seen from the bar charts in the previous text. In order to more comprehensively evaluate whether the image is distorted before and after being super-resolved, the FSIM was tested to supplement the results of the PSNR and SSIM. Overall, CMOSR effectively retains complex image details while significantly filtering out noise and stains, achieving "edge preservation and noise reduction". It has overall advantages in both subjective vision and objective evaluation and has great potential for obtaining the super-resolution of remote sensing images. In summary, the algorithm can well meet the needs of engineering applications for the super-resolution reconstruction of remote sensing images for high-speed space cameras.

### 5.2. Limitation

Despite possessing overall advantages over other methods, CMOSR also has certain shortcomings. For instance, while filtering out noise, it inevitably causes a certain degree of image smoothing, which will, to some extent, lose some texture details. Therefore, researching an effective extraction algorithm for fine textures to be integrated into the early dictionary training process is particularly important. Moreover, traditional reconstruction quality evaluation indicators such as the PSNR and SSIM can measure the reconstruction effect of super-resolution algorithms to a certain extent. However, for remote sensing images, which require noise components to be considered as a priority in super-resolution objects, they do not have complete evaluation adequacy and reliability. Therefore, it is necessary to establish a more targeted and persuasive evaluation index system in subsequent research.

## 6. Conclusions

This paper proposes a super-resolution reconstruction algorithm for edge-preserving denoising using sparse representation optimized by chaotic mapping (CMOSR). Based on the concept of compressed sensing and from the perspective of signal reconstruction, we use a method of joint training with high- and low-resolution dictionaries to obtain continuously updated dictionary pairs and the sparse representation coefficient. Joint training can make the high- and low-resolution dictionary pairs have the same sparse vector α, so as to make the model in the reconstruction process simpler and clearer and reduce the complexity of the solution. Relying on the inherent robustness of K-SVD, it effectively filters out noise while preserving the important structural information of the original image and introduces circle chaotic mapping to optimize the dictionary sequence update process. Using OMP to obtain sparse coefficients for upward reconstruction results in a twice-super-resolved image. In addition, to neutralize the smoothness caused by the denoising process, a local gradient is used as a constraint term to further highlight edge information. The final result is a high-quality super-resolved image, achieving an “edge-preserving denoising super-resolution”. Representative experiments have been conducted to demonstrate that the CMOSR proposed in this paper outperforms other super-resolution techniques in both subjective and objective evaluations. Experimental results show that the proposed algorithm CMOSR can significantly improve the SNR of remote sensing images by 2-4dB, which is the most concerned point of remote sensing image processing. The PSNR can reach an average of 35dB, the SSIM can be maintained above 0.91, the FSIM can be maintained above 0.95, and undesirable false details or artifacts are not generated during the reconstruction process. Furthermore, the integration of compressed sensing concepts offers novel super-resolution solutions for emerging application scenarios such as remote sensing images.

## Figures and Tables

**Table 1:** Comparisons of algorithms for target image over-scoring metrics.

	SRCNN	SRGAN	EDSR	ScSR	PSR	GigaGAN AI	CMOSR
MSE	3.79	91.41	113.33	2.57	616.02	550.43	3.00
PSNR	42.34 dB	28.52 dB	27.59 dB	44.04 dB	20.24 dB	20.72 dB	43.37 dB
SSIM	0.9891	0.9429	0.7858	0.9947	0.9192	0.8281	0.9866
FSIM	0.9964	0.9360	0.9271	0.9981	0.9798	0.8697	0.9908
Entropy	6.299	6.331	6.363	5.975	6.179	6.475	6.745

**Table 2:** Comparisons of various algorithms for aircraft fuselage image super-scoring metrics.

	SRCNN	SRGAN	EDSR	ScSR	PSR	GigaGAN AI	CMOSR
MSE	87.92	307.82	331.72	65.13	412.48	94.01	20.67
PSNR	28.69 dB	23.25 dB	22.92 dB	29.99 dB	21.98 dB	28.40 dB	34.98 dB
SSIM	0.9137	0.7110	0.6183	0.9305	0.9051	0.8467	0.9134
FSIM	0.9903	0.9433	0.9336	0.9923	0.9712	0.9624	0.9889
Entropy	6.724	6.698	6.547	6.497	6.190	6.428	6.768

**Table 3:** Comparisons of various algorithms for airport signage image superscoring metrics.

	SRCNN	SRGAN	EDSR	ScSR	PSR	GigaGAN AI	CMOSR
MSE	24.64	81.06	104.38	17.92	224.56	48.09	13.59
PSNR	34.21 dB	29.04 dB	27.95 dB	35.60 dB	24.62 dB	31.31 dB	36.80 dB
SSIM	0.9680	0.8950	0.7080	0.9736	0.9461	0.9301	0.9397
FSIM	0.9794	0.8859	0.8762	0.9794	0.9615	0.9462	0.9796
Entropy	5.328	5.303	5.308	5.075	4.912	5.046	5.226

**Table 4:** Comparisons of algorithms for global image superscoring metrics.

	SRCNN	SRGAN	EDSR	ScSR	PSR	GigaGAN AI	CMOSR
MSE	116.69	343.33	363.12	83.07	509.29	158.43	39.71
PSNR	27.46 dB	22.77 dB	22.53 dB	28.94 dB	21.06 dB	26.13 dB	32.14 dB
SSIM	0.9062	0.7154	0.6294	0.9263	0.9163	0.8547	0.9289
FSIM	0.9982	0.9698	0.96815	0.9959	0.9817	0.9862	0.9987
Entropy	7.277	7.238	7.274	7.262	6.808	7.156	7.326

**Table 5:** Comparisons of various algorithms for stadium image superscoring metrics.

	SRCNN	SRGAN	ScSR	GigaGAN AI	CMOSR
MSE	24.14	164.00	21.72	94.24	17.96
PSNR	34.30 dB	25.98 dB	34.76 dB	28.39 dB	35.59 dB
SSIM	0.9612	0.8097	0.9651	0.8699	0.9711
FSIM	0.9986	0.9596	0.9975	0.9844	0.9977
Entropy	7.493	7.487	7.490	7.488	7.499

**Table 6:** Comparisons of various algorithms for football field image superscoring metrics.

	SRCNN	SRGAN	ScSR	GigaGAN AI	CMOSR
MSE	58.83	275.02	44.39	88.91	38.66
PSNR	30.44 dB	23.74 dB	31.66 dB	28.64 dB	32.26 dB
SSIM	0.9208	0.6557	0.9365	0.8304	0.9439
FSIM	0.9959	0.9399	0.9944	0.9765	0.9986
Entropy	7.202	7.174	7.194	7.097	7.217

## Data Availability

Data are contained within the article.

## Abbreviations

The following abbreviations are used in this manuscript:

K-SVD	K-Singular Value Decomposition
OMP	Orthogonal Matching Pursuit
PSR	Pixel Super-Resolution
SRCNN	Super-Resolution Convolutional Neural Network
SRGAN	Super-Resolution Generative Adversarial Network
EDSR	Enhanced Deep Residual Networks
ScSR	Super-Resolution Via Sparse Representation
AI	Artificial Intelligence
CMOSR	Chaotic Mapping to Optimize Sparse Representation

## References

[1] Tsai R.Y., Huang T.S. (1984). Multiframe image restoration and registration. Multiframe Image Restor. Regist..

[2] Irani M., Peleg S. (1991). Improving resolution by image registration. CVGIP Graph. Model. Image Process..

[3] Stark H., Oskoui P. (1989). High-resolution image recovery from image-plane arrays, using convex projections. JOSA A.

[4] Banham M.R., Katsaggelos A.K. (1997). Digital image restoration. IEEE Signal Process. Mag..

[5] Schultz R.R., Stevenson R.L. (1996). Extraction of high-resolution frames from video sequences. IEEE Trans. Image Process..

[6] Freeman W.T., Pasztor E.C., Carmichael O.T. (2000). Learning low-level vision. Int. J. Comput. Vis..

[7] Freeman W.T., Jones T.R., Pasztor E.C. (2002). Example-based super-resolution. IEEE Comput. Graph. Appl..

[8] Glasner D., Bagon S., Irani M. Super-resolution from a single image. Proceedings of the 2009 IEEE 12th International Conference on Computer Vision.

[9] Chang H., Yeung D.Y., Xiong Y. Super-resolution through neighbor embedding. Proceedings of the 2004 IEEE Computer Society Conference on Computer Vision and Pattern Recognition (CVPR 2004).

[10] Hu H., Zhang J., Li B., Xu Z. (2021). Compressive sensing image reconstruction algorithm based on optimized sparse representation. Semicond. Optoelectron..

[11] Yang J., Wright J., Huang T.S., Ma Y. (2010). Image super-resolution via sparse representation. IEEE Trans. Image Process..

[12] Mallat S.G., Zhang Z. (1993). Matching pursuits with time-frequency dictionaries. IEEE Trans. Signal Process..

[13] Pati Y.C., Rezaiifar R., Krishnaprasad P.S. Orthogonal matching pursuit: Recursive function approximation with applications to wavelet decomposition. Proceedings of the 27th Asilomar Conference on Signals, Systems and Computers.

[14] Needell D., Vershynin R. (2009). Uniform uncertainty principle and signal recovery via regularized orthogonal matching pursuit. Found. Comput. Math..

[15] Donoho D.L., Tsaig Y., Drori I., Starck J.L. (2012). Sparse solution of underdetermined systems of linear equations by stagewise orthogonal matching pursuit. IEEE Trans. Inf. Theory.

[16] Sun G., Zhou Y., Wang Z., Dang W., Li Z. (2012). Sparsity adaptive compressive sampling matching pursuit algorithm based on compressive sensing. J. Comput. Inf. Syst..

[17] Jost P., Vandergheynst P., Frossard P. (2006). Tree-based pursuit: Algorithm and properties. IEEE Trans. Signal Process..

[18] La C., Do M.N. Tree-based orthogonal matching pursuit algorithm for signal reconstruction. Proceedings of the 2006 International Conference on Image Processing.

[19] Tan J., Cai M., Zhu X., Ge X. (2021). Super-resolution image reconstruction based on local structure similarity and sparse representation. J. Hefei Univ. Technol. (Nat. Sci.).

[20] Shen Y., Liu C., Yang Q. (2021). Super-Resolution Image Reconstruction Algorithm Using Sparse Features in Subspace. Comput. Eng. Appl..

[21] Dong C., Loy C.C., He K., Tang X. (2014). Learning a deep convolutional network for image super-resolution. Proceedings of the Computer Vision–ECCV 2014: 13th European Conference.

[22] Dong C., Loy C.C., Tang X. (2016). Accelerating the super-resolution convolutional neural network. Proceedings of the Computer Vision–ECCV 2016: 14th European Conference.

[23] Shi W., Caballero J., Huszár F., Totz J., Aitken A.P., Bishop R., Rueckert D., Wang Z. Real-time single image and video super-resolution using an efficient sub-pixel convolutional neural network. Proceedings of the IEEE conference on Computer Vision and Pattern Recognition.

[24] Liu F., Yang X., De Baets B. (2023). A deep recursive multi-scale feature fusion network for image super-resolution. J. Vis. Commun. Image Represent..

[25] He K., Zhang X., Ren S., Sun J. Deep residual learning for image recognition. Proceedings of the IEEE Conference on Computer Vision and Pattern Recognition.

[26] Kim J., Lee J.K., Lee K.M. Accurate image super-resolution using very deep convolutional networks. Proceedings of the IEEE Conference on Computer Vision and Pattern Recognition.

[27] Lim B., Son S., Kim H., Nah S., Mu Lee K. Enhanced deep residual networks for single image super-resolution. Proceedings of the IEEE Conference on Computer Vision and Pattern Recognition Workshops.

[28] Haris M., Shakhnarovich G., Ukita N. Deep back-projection networks for super-resolution. Proceedings of the IEEE Conference on Computer Vision and Pattern Recognition, Salt Lake City.

[29] Zhang Y., Li K., Li K., Wang L., Zhong B., Fu Y. Image super-resolution using very deep residual channel attention networks. Proceedings of the European Conference on Computer Vision (ECCV).

[30] Tai Y., Yang J., Liu X. Image super-resolution via deep recursive residual network. Proceedings of the IEEE Conference on Computer Vision and Pattern Recognition.

[31] Jiang K., Wang Z., Yi P., Jiang J. (2020). Hierarchical dense recursive network for image super-resolution. Pattern Recognit..

[32] Lin D., Xu G., Xu W., Wang Y., Sun X., Fu K. (2020). SCRSR: An efficient recursive convolutional neural network for fast and accurate image super-resolution. Neurocomputing.

[33] Li Z., Liu Y., Chen X., Cai H., Gu J., Qiao Y., Dong C. Blueprint separable residual network for efficient image super-resolution. Proceedings of the IEEE/CVF Conference on Computer Vision and Pattern Recognition.

[34] Gendy G., Sabor N., Hou J., He G. Mixer-based local residual network for lightweight image super-resolution. Proceedings of the IEEE/CVF Conference on Computer Vision and Pattern Recognition.

[35] Ledig C., Theis L., Huszár F., Caballero J., Cunningham A., Acosta A., Aitken A., Tejani A., Totz J., Wang Z. Photo-realistic single image super-resolution using a generative adversarial network. Proceedings of the IEEE Conference on Computer Vision and Pattern Recognition.

[36] Wang X., Yu K., Wu S., Gu J., Liu Y., Dong C., Qiao Y., Change Loy C. Esrgan: Enhanced super-resolution generative adversarial networks. Proceedings of the European Conference on Computer Vision (ECCV) Workshops.

[37] Kang M., Zhu J.Y., Zhang R., Park J., Shechtman E., Paris S., Park T. Scaling up gans for text-to-image synthesis. Proceedings of the IEEE/CVF Conference on Computer Vision and Pattern Recognition.

[38] Karras T., Laine S., Aittala M., Hellsten J., Lehtinen J., Aila T. Analyzing and improving the image quality of stylegan. Proceedings of the IEEE/CVF Conference on Computer Vision and Pattern Recognition.

[39] Wang Z., Zhang Z., Zhang X., Zheng H., Zhou M., Zhang Y., Wang Y. Dr2: Diffusion-based robust degradation remover for blind face restoration. Proceedings of the IEEE/CVF Conference on Computer Vision and Pattern Recognition.

[40] Lee R., Li R., Venieris S., Hospedales T., Huszár F., Lane N.D. Meta-learned kernel for blind super-resolution kernel estimation. Proceedings of the IEEE/CVF Winter Conference on Applications of Computer Vision.

[41] Zhou H., Zhu X., Zhu J., Han Z., Zhang S.X., Qin J., Yin X.C. Learning Correction Filter via Degradation-Adaptive Regression for Blind Single Image Super-Resolution. Proceedings of the IEEE/CVF International Conference on Computer Vision.

[42] Weng S.Y., Yuan H., Xu Y.S., Huang C.C., Chiu W.C. Best of Both Worlds: Learning Arbitrary-Scale Blind Super-Resolution via Dual Degradation Representations and Cycle-Consistency. Proceedings of the IEEE/CVF Winter Conference on Applications of Computer Vision.

[43] Lee H., Kim J., Kim J., Jeon P., Lee S.A., Kim D. (2021). Noniterative sub-pixel shifting super-resolution lensless digital holography. Opt. Express.

[44] Gao Y., Cao L. (2021). Generalized optimization framework for pixel super-resolution imaging in digital holography. Opt. Express.

[45] Zhang Y., Huang Y., Zhang Y., Liu S., Luo J., Zhou X., Yang J., Jakobsson A. (2023). High-throughput hyperparameter-free sparse source location for massive TDM-MIMO radar: Algorithm and FPGA implementation. IEEE Trans. Geosci. Remote. Sens..

[46] Stoica P., Babu P., Li J. (2010). New method of sparse parameter estimation in separable models and its use for spectral analysis of irregularly sampled data. IEEE Trans. Signal Process..

[47] Cheng G., Han J., Zhou P., Guo L. (2014). Multi-class geospatial object detection and geographic image classification based on collection of part detectors. ISPRS J. Photogramm. Remote Sens..

[48] Sun X., Wang P., Yan Z., Xu F., Wang R., Diao W., Chen J., Li J., Feng Y., Xu T. (2022). FAIR1M: A benchmark dataset for fine-grained object recognition in high-resolution remote sensing imagery. ISPRS J. Photogramm. Remote Sens..

